# Supplement strategies for infertility in overweight women: Evidence and legal insights

**DOI:** 10.1515/med-2025-1293

**Published:** 2025-09-25

**Authors:** Giuseppe Gullo, Eleonora Conti, Valentina Billone, Elena Chitoran, Karolina Kowalcze, Robert Krysiak, Alberto Vaiarelli, Romualdo Sciorio, Stamatios Petousis, Yuliia Kotlik, Antonio Perino, Gaspare Cucinella, Susanna Marinelli, Lina De Paola

**Affiliations:** Department of Obstetrics and Gynaecology, AOOR Villa Sofia – Cervello, University of Palermo, Palermo, Italy; General Surgery and Surgical Oncology Department I, Bucharest Institute of Oncology “Al. Trestioreanu”, 022328, Bucharest, Romania; Department of Pathophysiology, Faculty of Medicine, Academy of Silesia, 40-555, Katowice, Poland; Department of Pediatrics in Bytom, Faculty of Health Sciences in Katowice, Medical University of Silesia, 41-902, Bytom, Poland; Department of Internal Medicine and Clinical Pharmacology, Medical University of Silesia, 40-752, Katowice, Poland; IVIRMA Global Research Alliance, Genera, Clinica Valle Giulia, Rome, Italy; Fertility Medicine and Gynaecological Endocrinology Unit, Department Woman Mother Child, Lausanne University Hospital, 1011, Lausanne, Switzerland; 2nd Department of Obstetrics and Gynaecology, Aristotle University of Thessaloniki, 541-24, Thessaloniki, Greece; School of Law, Polytechnic University of Marche, 60121 Ancona, Italy; Department of Anatomical, Histological, Forensic and Orthopedic Sciences, Sapienza University of Rome, 00161, Rome, Italy

**Keywords:** reproductive dysfunction, nutraceutical, obese female patients, medico-legal implications

## Abstract

**Background:**

Infertility is a multifactorial condition that affects both men and women and is influenced by various factors, including overweight and obesity. These conditions, especially in women with polycystic ovary syndrome (PCOS), are strongly associated with hormonal and metabolic imbalances that can impair fertility. Targeted nutritional interventions, such as nutraceutical supplementation, may offer support in improving reproductive outcomes.

**Methods:**

A narrative review was conducted using PubMed, focusing on publications from the past 12 years with the keywords “nutraceutical,” “overweight,” and “infertility.” The review aimed to identify the main nutraceuticals used in managing infertility and to highlight the importance of a personalized approach tailored to individual patient characteristics.

**Results:**

Nutraceuticals may represent a safe and cost-effective adjunctive strategy to support fertility in overweight patients, particularly in those with PCOS. Evidence suggests that their effectiveness increases when integrated into a personalized treatment plan based on individual needs and clinical profiles.

**Conclusions:**

This review offers an updated overview of nutraceutical use in overweight individuals with infertility, outlining both benefits and limitations. It also addresses the often-overlooked medico-legal aspects of prescribing nutraceuticals, emphasizing the need for ethical and legal awareness when incorporating these interventions into clinical practice.

## 1 Introduction

Infertility is a condition characterized by the inability of a couple to conceive after at least 12 months of regular, unprotected sexual intercourse [1]. It is a growing global concern, affecting approximately one in seven couples. The causes of infertility are multifactorial and can be classified into male-only factors (30% of cases), combined male and female factors (20%) [2], female-only factors (35%) [3], and unexplained causes (15%) [4,5]. This classification highlights the complexity of infertility and underscores the need for personalized treatment approaches. Among the many causes of female infertility, ovulatory disorders, tubal pathologies, and chronic conditions are the most common [6]. One of the leading causes of infertility in women is polycystic ovary syndrome (PCOS), a condition affecting a significant number of women during their reproductive years. PCOS is recognized as one of the most prevalent endocrine disorders in women of reproductive age, characterized primarily by a combination of hyperandrogenism (elevated male hormone levels), oligo-/anovulation (infrequent or absent ovulation), and the presence of multiple small cysts on the ovaries, as observed on ultrasound [7]. A major challenge for women with PCOS is the creation of a vicious cycle between obesity and infertility, in which obesity exacerbates hormonal imbalances, such as insulin resistance and hyperandrogenism, which in turn impair ovulation and fertility. Moreover, the hyperandrogenism characteristic of PCOS often promotes the accumulation of visceral fat, further disrupting the hormonal environment and perpetuating the cycle of worsening symptoms [8,9]. This interplay highlights the need for effective interventions that address both metabolic and reproductive issues in managing PCOS-related infertility.

The relationship between obesity and infertility is well-established, with numerous studies demonstrating that excess body weight can significantly impact fertility. Obesity alters the hypothalamic–pituitary–gonadal (HPG) axis, a crucial hormonal pathway that regulates reproductive function. Increased adipose tissue results in higher aromatization of androgens (male hormones) into estrogens (female hormones), disrupting the hormonal balance within the reproductive system [10]. This hormonal imbalance triggers a negative feedback loop on the HPG axis, leading to reduced gonadotropin production and impairing ovulation and menstrual cycles [11,12]. As a result, women with obesity often experience menstrual irregularities and reduced fertility.

In addition to hormonal disruptions, obesity has been shown to negatively affect oocyte quality and endometrial receptivity – two critical factors for successful conception. The impact of obesity on oocyte quality presents challenges for both natural conception and assisted reproductive technologies (ART), such as *in vitro* fertilization (IVF) [7]. Several studies have demonstrated that a body mass index (BMI) greater than 25 kg/m² significantly reduces the success of ART. Women with higher BMI levels tend to have lower-quality eggs and embryos, requiring higher doses of gonadotropins to stimulate ovulation and longer durations of ovulation induction. Additionally, obesity is associated with a higher risk of complications during ART procedures, such as oocyte retrieval and embryo transfer, as well as an increased likelihood of miscarriage following conception. The growing body of evidence linking obesity to infertility has raised questions about how nutrition can be utilized to improve fertility outcomes, particularly in overweight or obese individuals. Research has shown that dietary patterns play a crucial role in reproductive health. Diets high in fatty acids, refined carbohydrates, and added sugars have been found to negatively impact fertility by exacerbating insulin resistance, inflammation, and hormonal imbalances. Conversely, a balanced diet that includes fiber, omega-3 fatty acids, proteins, vitamins, and minerals has been associated with improved fertility outcomes.

These nutrients are essential for maintaining hormonal balance, reducing inflammation, and promoting healthy ovarian function [4]. Given the positive effects of nutrition on fertility, nutraceutical supplementation has emerged as a potential strategy for enhancing reproductive outcomes, particularly in overweight or obese patients. Nutraceuticals, which include vitamins, minerals, fatty acids, and plant-based compounds, are thought to support fertility by addressing dietary deficiencies or imbalances. By filling specific nutritional gaps, nutraceuticals may help improve metabolic and hormonal balance, thereby enhancing fertility in overweight and obese individuals.

This review aims to analyze the benefits and limitations of nutraceutical supplementation in the management of infertility, with a particular focus on patients who are overweight or obese. Given the well-established correlation between obesity and PCOS, numerous authors, following a review of the literature, frequently include PCOS patients in their analyses of nutraceutical use in overweight women with infertility; accordingly, the present review also includes patients with PCOS within its scope of investigation. It will explore how specific dietary supplements may influence reproductive function, hormonal balance, and metabolic health, while also addressing the current scientific evidence, safety concerns, and gaps in knowledge related to their use in this specific population. Therefore, we examine the mechanisms through which various nutraceuticals, such as antioxidants, micronutrients, amino acids, and plant-derived extracts, may improve reproductive outcomes by targeting oxidative stress, insulin resistance, inflammation, and endocrine dysfunction. Furthermore, this review discusses the current clinical evidence supporting their efficacy and safety and identifies potential interactions or contraindications. We try to provide a perspective that can support clinical decision-making and guide future research in this evolving field.

## 2 Materials and methods

Literature research was conducted to investigate nutraceutical supplementation in overweight infertile patients. A comprehensive search was performed in PubMed and Scopus, covering the period from 2014 to 2025. The keywords used were “nutraceutical,” “overweight,” and “infertility.” A total of 63 papers were identified, and only those written in English were included. Initially, the titles and abstracts of all identified papers were reviewed. Papers were first analyzed based on their titles and abstracts to assess their potential relevance to the research topic. If a study appeared to be potentially useful, it was then considered for full-text reading and detailed analysis. Inclusion criteria were articles that were pertinent to the research topic, involved only human data, and consisted of either reviews or studies with empirical data. Only articles related to the most commonly used nutraceuticals were included in the research, while acknowledging the infinite variety of nutraceutical products that may be potentially used.

**Figure 1:**
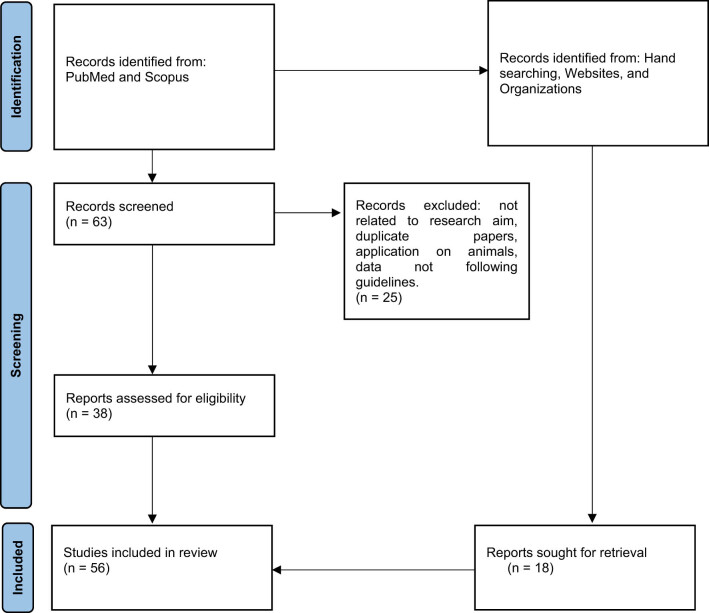
PRISMA flow chart.

Ultimately, 38 articles were selected that addressed the role of nutraceutical supplementation in overweight infertile patients (Figure 1). Additionally, the relevant forensic literature was reviewed to assess the medico-legal implications of such supplementation.

This review aims to analyze the benefits of nutraceutical supplementation in improving the fertility of overweight patients, such as those with PCOS, while also identifying potential gaps in the existing research.

## 3 Results

### 3.1 Role of vitamin D

Many authors have focused on evaluating the role of vitamin D supplementation in cases of infertility, as there appears to be a correlation between vitamin D deficiency and infertility. In 2015, a review by Dabrowski et al. [13] examined the role of vitamin D in the treatment of infertility in patients with PCOS, uterine fibroids, or male-factor infertility, including 235 articles. This review concluded that vitamin D supplementation is recommended in infertility therapy for both partners, noting that couples with a serum vitamin D concentration around 50 nmol/L have a higher chance of conception. Supplementation was particularly recommended for obese, insulin-resistant women with low anti-Müllerian hormone (AMH) levels. Additionally, no adverse effects were reported with an intake of up to 10,000 IU/day.

In 2019, Bosdou et al. [14] conducted a review on the impact of vitamin D deficiency and obesity on male and female infertility. They examined both observational and interventional studies and demonstrated that vitamin D deficiency and obesity negatively affect fertility in both genders. However, the evidence from interventional studies on vitamin D supplementation was limited, as these studies had small sample sizes and significant variability in dosage and duration.

Some authors have investigated the impact of vitamin D supplementation on serum levels of AMH, a key ovarian biomarker for folliculogenesis and ovarian reserve. Moridi et al. [15] conducted a systematic review in 2019 analyzing 18 observational and 6 interventional studies on the association between vitamin D and AMH. They concluded that the relationship between vitamin D and AMH is complex and non-linear.

Specifically, the effects of vitamin D on AMH appear to depend on the ovulatory status of the patient. In patients with PCOS, vitamin D seems to decrease AMH levels, while in those without PCOS, it appears to increase AMH levels. Since AMH is abnormally elevated in patients with PCOS, vitamin D supplementation seems to improve folliculogenesis in these individuals.

Another study conducted by Lerchbaum et al. [16] was a single-center, double-blind trial performed from 2011 to 2017 at the Medical University of Graz, Austria. The study included 180 women with PCOS and 150 without PCOS, all with serum vitamin D concentrations <75 nmol/L. The markers evaluated included AMH, LH, follicle-stimulating hormone (FSH), estradiol, dehydroepiandrosterone sulfate, and androstenedione. The study showed that, in women with PCOS, vitamin D supplementation had a significant effect on FSH values and the FSH/LH ratio but no effect on AMH levels. Furthermore, no significant effects were observed in women without PCOS.

The effects of vitamin D supplementation on AMH levels, metabolic profiles, and gene expression related to lipid and insulin metabolism in PCOS patients undergoing IVF have also been studied. Dastorani et al. [17] conducted a randomized, double-blind study on 40 infertile women aged 18 to 40 with a diagnosis of PCOS undergoing IVF. One group received 50,000 IU of vitamin D weekly for 8 weeks, while the other group received a placebo. Vitamin D supplementation demonstrated beneficial effects on lipid and insulin metabolism in these patients. Vitamin D appears to play a protective role by modulating blood pressure in hypertensive disorders, improving insulin sensitivity in gestational diabetes, and contributing to the regulation of cell proliferation and differentiation in carcinogenesis [18,19]. These findings reveal conflicting results regarding the correlation between vitamin D levels and AMH. However, such discrepancies may be attributed to factors such as variations in vitamin D dosage, differences in study design, baseline vitamin D status of the participants, the presence of underlying metabolic disorders, or even the sample size of the studies.

Nonetheless, most of the studies included in our analysis reported a positive correlation between vitamin D and AMH levels.

### 3.2 Role of antioxidants: Omega-3 and vitamin E

Various studies have demonstrated that oxidative stress plays a significant role in infertility in patients with PCOS. Oxidative stress refers to an imbalance between the production of free radicals and the body’s ability to neutralize them with antioxidants [20]. This imbalance has been shown to increase the production of androgens and insulin, while also contributing to poorer ovarian follicle quality [21]. It has been observed that patients with PCOS exhibit elevated levels of oxidative stress indices, such as malondialdehyde [22]. In this context, omega-3 fatty acids are considered one of the primary nutritional supplements for counteracting oxidative stress in PCOS patients. Omega-3s are polyunsaturated fatty acids found predominantly in vegetable and marine oils [23], and they have beneficial effects on various health conditions. For example, omega-3s have been shown to significantly impact obesity, with higher levels of omega-3s resulting in a reduction in fat mass [24]. Since obese patients with PCOS tend to have more severe hyperandrogenism, a higher incidence of anovulatory cycles, and oligomenorrhea, they are at an increased risk of infertility. As a result, several studies have explored the potential benefits of omega-3 supplementation in these patients.

Nadjarzadeh et al. [25] conducted a randomized, double-blind study in 2015 on 84 obese infertile patients with PCOS, investigating the effects of omega-3 intake on adiponectin, LH, FSH, and visfatin levels. The study found that these patients exhibited elevated visfatin levels, an adipokine produced by visceral fat and associated with insulin resistance, which plays a role in the pathogenesis of PCOS. Additionally, adiponectin levels were reduced, while adiponectin is an adipokine that improves insulin sensitivity and exerts anti-inflammatory effects. The study demonstrated that omega-3 supplementation led to an increase in adiponectin, beneficial effects on LH concentrations, and an improved LH/FSH ratio. However, no effects were observed on visfatin, FSH, prolactin, or BMI.

The potential impact of omega-3 supplementation on spontaneous conception has also been studied. In 2022, Stanhiser et al. [26] analyzed data from a study conducted between 2008 and 2015 involving 1,036 women aged 30–44, all attempting conception for less than three months without a history of infertility. The study concluded that women who took omega-3 supplements had a 1.51 times greater chance of spontaneous conception compared to those who did not.

In addition to omega-3, vitamin E is another potent antioxidant known to have significant effects on women’s reproductive processes. A 2022 review by Amin et al. [27] assessed the role of vitamin E supplementation in female reproduction, focusing on its effects on fertility, reproductive hormone levels, and assisted reproductive technologies. Although the exact mechanism by which vitamin E acts is not fully understood, the review suggests that it has promising potential to improve fertility rates and overall reproductive health in women.

Given that both omega-3 and vitamin E are strong antioxidants, a study also explored the potential combined effects of these supplements in infertile patients with PCOS and a BMI greater than 25. In a randomized, double-blind study [28], 62 patients were assigned to either the intervention group (group A) or the control group (group B). Group A received co-supplementation of 2 g of omega-3 and 400 IU of vitamin E for 8 weeks, while group B received a placebo for the same duration. The study evaluated total antioxidant capacity, glutathione levels, catalase activity, and malondialdehyde concentrations before and after the supplementation period. The results showed that co-supplementation with omega-3 and vitamin E led to a significant increase in total antioxidant capacity (1.15 ± 0.93 vs 0.6 ± 0.72; *P* < 0.001), catalase activity (1.19 ± 1.06 vs 0.12 ± 0.36; *P* < 0.001), and glutathione levels (1.5 ± 1.06 vs 0.23 ± 1.43; *P* = 0.028), along with a significant reduction in malondialdehyde levels (0.34 ± 0.32 vs 0.57 ± 2.20; *P* = 0.008) compared to the placebo group.

### 3.3 Role of supplementation with probiotics and synbiotics

In patients with PCOS and BMI > 25, a strategy to address obesity and improve reproductive outcomes involves dietary supplementation with probiotics and synbiotics. Probiotics are live microorganisms that confer health benefits to the host, while synbiotics are dietary supplements that combine probiotics with prebiotics (non-digestible food ingredients that promote the growth of probiotics). Their beneficial effects are attributed to their ability to modulate the intestinal microbiota [29,30,31,32].

Chudzicka-Strugała et al. [33] conducted a randomized, double-blind study in 2021 involving 65 patients diagnosed with PCOS and a BMI of >25, recruited from the Reproductive Endocrinology & Infertility Clinical Services at Poznan University of Medical Sciences. Participants were randomly assigned to receive either synbiotic supplementation or a placebo. The synbiotic supplement included several probiotics: two strains of *Bifidobacterium lactis*, *Lactobacillus acidophilus*, *Lactobacillus paracasei*, *Lactobacillus plantarum*, *Lactobacillus salivarius*, and *Lactobacillus lactis*. The study evaluated changes in BMI and testosterone levels.

The synbiotic group exhibited an 8% reduction in BMI, significantly greater than the 5% reduction observed in the placebo group (*P* = 0.03). Additionally, a reduction in total testosterone levels was observed in 90% of the women in the synbiotic group compared to 53% in the placebo group, with a significant difference between the groups (*P* = 0.008). Testosterone levels decreased by 32% in the synbiotic group, compared to only a 6% decrease in the placebo group.

This study supports the hypothesis that synbiotic supplementation can enhance the effects of diet and exercise in promoting weight loss and reducing hyperandrogenism in obese patients with PCOS.

### 3.4 Role of inositols

Inositols are a group of cyclic polyols with a six-carbon ring, which have been shown to play an important role in insulin-activated signaling pathways [34,35,36]. The most important isoforms are myo-inositol and d-chiro-inositol. These compounds are considered insulin-sensitizing, and therefore, their deficiency leads to insulin resistance. For this reason, their role has been studied in patients with PCOS [37,38,39].

A review conducted by Coldebella et al. in 2022 [38] analyzed the role of inositol treatment in patients with PCOS, demonstrating a reduction in hyperinsulinemia and an improvement in the metabolic and ovulatory characteristics of these patients. Beneficial effects on oocytes and ovarian quality were also shown in patients undergoing ART. However, this review did not determine which of the two isoforms is the most effective or what the optimal dosage is.

Some animal studies have also evaluated the effect of myo-inositol and d-chiro-inositol in the treatment of PCOS and have shown that a 40:1 ratio of the two positively modulates the steroidogenic pathway of the ovarian theca cells and increases the concentration of FSH receptors in granulosa cells [35].

Inositols have often been combined in PCOS treatment [40,41] with alpha-lactalbumin [42], a milk whey globular protein produced by the epithelial cells of the mammary gland, which has prebiotic, mucoprotective, and anti-inflammatory actions. Due to its prebiotic effect, alpha-lactalbumin appears to promote the intestinal absorption of certain substances, including inositols, thereby enhancing their effects on PCOS [43].

### 3.5 Other nutraceuticals

Another promising nutraceutical is melatonin, a low molecular weight hormone that modulates multiple metabolic pathways in humans, including the regulation of circadian rhythms, reproductive mechanisms, and immune responses [44]. In the context of female fertility, supplementation with melatonin has been shown to improve oocyte and embryo quality, as well as luteal function [45,46]. These findings support the growing interest in melatonin as a promising adjunct in the treatment of female infertility [47]. It has also been demonstrated that melatonin supplementation, particularly when combined with magnesium, has beneficial effects in patients with PCOS, improving parameters such as hirsutism, BMI, waist circumference, and serum levels of TNF-alpha [48]. Moreover, the combined administration of myo-inositol and melatonin has been compared with myo-inositol alone in PCOS patients undergoing IVF, and it was observed that the addition of 3 mg of melatonin to 400 mg of myo-inositol enhances both oocyte and embryo quality [49].

Coenzyme Q10 (CoQ10) is a powerful antioxidant and free radical scavenger that acts primarily within mitochondria – organelles essential for energy metabolism and highly susceptible to oxidative damage [50]. CoQ10 plays a protective role in female gametes against oxidative stress, with studies showing that its concentration in follicular fluid declines significantly with age [51,52]. Furthermore, CoQ10 supplementation has been associated with improvements in ovarian response, oocyte quality, and embryo development, particularly in women with diminished ovarian reserve [53].

Selenium is another compound – specifically a micronutrient – with significant effects in women with PCOS, particularly as an adjuvant to pharmacological treatment with metformin. It has been shown to improve fasting blood glucose levels, reduce insulin resistance, and lower free testosterone levels [54,55]. Although selenium supplementation in infertile PCOS patients undergoing IVF has shown beneficial effects on glycemic control, it does not appear to influence pregnancy rates, lipid profile, total antioxidant capacity, or total glutathione levels [56].

From our research, we have created Table 1 that summarizes the most significant data of the most used nutraceuticals in clinical practice.

**Table 1:** Most prescribed nutraceuticals

Nutraceutical	Main function	Fertility benefits	Dosage
Vitamin D	Regulation of the immune system and hormonal balance	Improves oocyte quality, insulin sensitivity, endometrial receptivity, and embryo quality	20,000–50,000 UI/week
Omega-3 (EPA and DHA)	Essential fatty acids for the body	Improves oocyte quality and sperm motility and has a significant impact on obesity	500–1,500 mg daily
Vitamin E	Antioxidant that protects from radiation and oxidative stress	Improves semen quality and ovarian function. Enhances the endometrial environment and improves endometrial thickness	400UI twice daily
Myo-Inositol	Helps insulin function and regulates ovulation	Supports oocyte quality, useful in women with PCOS	2 g twice a day
Alpha-Lipoic Acid	Powerful antioxidant that supports metabolism and reduces inflammation	Improves oocyte and sperm quality, useful in patients with PCOS and insulin resistance	600 mg twice daily or 800 mg daily
Melatonin	A low molecular weight hormone that modulates the regulation of circadian rhythms, reproductive mechanisms, and immune responses	Improves oocyte, embryo quality, and luteal function	3 mg daily
Coenzyme Q10	A powerful antioxidant and free radical scavenger that acts primarily within mitochondria	Improves ovarian response, oocyte quality, and embryo development	100–200 mg daily
Selenium	Improves fasting blood glucose levels and reduces insulin resistance	Increases AMH index and antral follicle count	200 ng/day

### 3.6 Legal framework

From a medico-legal perspective, the use of nutraceutical therapies in overweight or obese individuals, particularly those affected by single or multiple systemic or solely gynecological conditions, involves numerous and complex issues that go beyond the simple prescription of a supplement. Many nutraceuticals are prescribed or purchased independently by patients as potentially effective solutions for weight management and the improvement of metabolic and aesthetic conditions; however, the scientific evidence supporting their efficacy is often limited or even conflicting.

Therefore, the use of these products should only occur in the presence of sufficiently robust clinical data or with regulatory approval comparable to that of traditional pharmaceuticals, thus avoiding medical problems resulting from their unverified safety [57]. The possibility of side effects, drug interactions, or the lack of adequate monitoring for some of these products could lead to significant direct or indirect harm to the patient. The risks associated with unverified and unsafe products cannot be ignored. In extreme cases, some nutraceuticals or substances contained within them can be used as additives in the production of synthetic drugs, such as in the case of “bath salts” [58,59]. Preventing the expansion of this market begins with proper education and appropriate prescriptions, not only of medications but also of nutraceutical supplements, to avoid their potential conversion into dangerous and illegal substances that end up in the black market for illicit purposes [60]. It is crucial that nutraceuticals be prescribed carefully, also for public safety reasons, and that healthcare professionals educate patients on the correct use of these products and the dangers of their abuse or the use of unregulated or unauthorized substances [61,62]. Proper regulation and transparency in prescriptions can also prevent the overproduction of unnecessary or misused substances [63].

From a medico-legal standpoint, healthcare professionals are required to ensure the scientific validity of any nutraceutical treatment. This choice should be based on a careful evaluation of the risk-benefit ratio, in line with available scientific evidence and sector guidelines, while always adhering to the principle of primum non nocere (first, do not harm) [64] and the legality of the medical treatment. The professional responsibility of prescribing healthcare providers also extends to ensuring the proper information is given to the patient [65]. Just as with other prescribed medications or vitamins, healthcare providers must inform patients adequately about the potential side effects and limitations of the treatment to avoid any form of negligence. Informed consent, as in many other areas of medicine, cannot be overlooked and must always be based on clarity of information, transparency of data, simplicity in conveying scientific information, and up-to-date medical knowledge.

Moreover, the prescription of nutraceuticals should be contextualized within a global therapeutic plan that considers the patient’s specific health conditions. As previously mentioned, the prescription of these substances should follow the principle of personalized medicine, especially in the presence of significant systemic comorbidities such as diabetes, hypertension, or dyslipidemia, which could also affect the efficacy and safety of nutraceutical intake. Close attention must be given to the consequences or benefits for fertility [7,66,67,68]. This aspect must be clearly communicated to patients, particularly to avoid misunderstandings and potential disputes. The lack of stringent regulation for nutraceuticals, compared to pharmaceuticals, should lead to greater caution on the part of the prescribing physician, particularly in the continuous monitoring of effects and management of potential complications. In the case of harm arising from the use of such therapies, the physician could face legal disputes related to professional responsibility based on negligence. This could especially occur in cases where the treatment is not adequately and scientifically justified, if risks were not properly considered, or if sufficient information was not provided. It is also important to note that even when adequate information is provided, it must always be well documented and verifiable, as there are cases in professional healthcare liability where legal disputes are based on the lack of documentation proving that information was given [69]. Proper documentation of the decision-making process and transparency in communications with the patient are, therefore, crucial to preventing potential legal disputes and protecting the healthcare professional’s legal responsibility, as well as safeguarding the patient’s right to health and self-determination.

Therefore, to enhance clarity, we conducted a concise comparison between the regulatory frameworks of the European Union (EU) and the United States regarding the use of nutraceuticals in gynecology, particularly with reference to the medico-legal context, highlighting substantial differences.

In the EU, nutraceuticals are generally classified as food supplements and are subject to the regulatory framework governing functional foods. Conversely, in the United States, the term falls under dietary supplements, regulated by the Dietary Supplement Health and Education Act (DSHEA) of 1994 [70].

The main European regulations include Regulation (EC) No. 1924/2006 (nutritional and health claims) [71], Regulation (EU) No. 1169/2011 (labeling requirements) [72], and Directive 2002/46/EC (food supplements) [73].

Any health claim (e.g., “supports female health”) must be evaluated and approved by EFSA (European Food Safety Authority). These supplements cannot replace conventional medical treatments and must not suggest curative effects, but rather be positioned as adjunctive aids to health [74].

In the United States, the federal DSHEA permits manufacturers to market dietary supplements without being required to demonstrate their safety or effectiveness beforehand [75]; nutraceuticals are regulated by the FDA as dietary supplements but are not subject to the same premarket approval processes as pharmaceuticals [76]. The manufacturer is entirely responsible for product safety and the truthfulness of any benefit-related statements. The FDA may only intervene in post-marketing, once the product is already available to consumers [77].

Moreover, if a health claim is made, it’s legally required to include the disclaimer “This statement has not been evaluated by the FDA. This product is not intended to diagnose, treat, cure, or prevent any disease.”

In Europe, the use of nutraceuticals for medical purposes is not typically covered by public health insurance. Physicians may face professional liability if they recommend a product with unproven pharmacological efficacy for therapeutic purposes.

In the United States, physicians have greater prescriptive freedom, yet they can still be held legally accountable for adverse outcomes resulting from inappropriate use of nutraceuticals or failure to recommend recognized conventional treatments [74].

The fundamental differences between the EU and the United States are therefore summarized in Table 2.

**Table 2:** Summary of key differences

Aspect	EU	The United States
Pre-market evaluation	Rigorous (EFSA approval required)	Minimal (FDA acts post-market)
Manufacturer liability	High, moderated by EFSA authorization	Full responsibility, no pre-approval control
Physician liability	High, especially in cases of unproven claims	High, particularly if informed consent is lacking
Patient protection	Strongly oriented toward the precautionary principle	More focused on individual freedom

Therefore, the EU adopts a more restrictive and precautionary approach, requiring scientific evidence for all health claims and holding physicians accountable for nutraceutical prescriptions. In contrast, the United States follows a more liberal model, characterized by limited initial oversight and regulation, but with significant legal consequences in cases of adverse effects or misleading claims.

## 4 Discussion

Our research suggests that overweight infertile patients may benefit from nutraceutical supplementation, particularly when addressing underlying conditions such as PCOS, which is commonly diagnosed in these patients. The prevalence of PCOS among women with infertility underscores the need for targeted nutritional interventions to improve fertility outcomes. One key component in this approach is vitamin D, a secosteroid hormone with crucial roles in both metabolic and reproductive processes [78].

In women with PCOS, vitamin D supplementation has shown significant potential in improving various aspects of reproductive health. Vitamin D is involved in several physiological mechanisms essential for fertility. It helps enhance insulin sensitivity, which is a critical concern for many women with PCOS who experience insulin resistance. Additionally, vitamin D has been shown to positively affect endometrial receptivity – an essential factor for embryo implantation – along with promoting proper oocyte (egg) development and embryo quality. These beneficial effects of vitamin D are primarily mediated through its receptors in ovarian granulosa cells, which are essential for ovarian function and overall reproductive health [79].

Another important aspect of vitamin D in fertility is its influence on ovarian reserve, commonly assessed by AMH levels [80,81]. AMH is produced by granulosa cells during the follicular phase of ovarian development and serves as a crucial marker of ovarian reserve. Research suggests that vitamin D plays a role in regulating AMH expression and serum levels. A deficiency in vitamin D may lead to disturbances in AMH production [82]. AMH binds to two types of receptors (type I and type II), and its interaction with type II receptors can inhibit follicular maturation and decrease ovarian sensitivity to FSH, which is vital for follicle development [83]. Vitamin D supplementation appears to counteract this effect by inhibiting the expression of the AMHR-II receptor, thus promoting follicular maturation and improving ovarian function [84,85]. Therefore, the assessment of vitamin D status may represent a valuable component in fertility protocols, particularly in patients with PCOS or borderline ovarian reserve. Appropriate vitamin D supplementation has the potential to enhance reproductive outcomes; yet, it requires individualized dosing strategies and regular monitoring of serum levels to ensure efficacy and safety. This interaction further supports the potential role of vitamin D as an adjuvant in hormonal treatment protocols, especially within clinically supervised and personalized reproductive care settings.

Despite the growing evidence supporting the benefits of vitamin D for PCOS patients, no universally accepted standard exists for the optimal serum levels of vitamin D in fertility treatment. The Endocrine Society’s guidelines suggest that vitamin D levels above 30 ng/mL are sufficient, levels between 20 and 29 ng/mL indicate insufficiency, and levels below 20 ng/mL signal deficiency. For PCOS patients with insulin resistance and low AMH levels, particularly those undergoing fertility treatments, the recommended dosage of vitamin D3 is 1,500–2,000 IU per day. This supplementation is thought to improve fertility outcomes by addressing both metabolic and reproductive dysfunctions in these patients [86].

In addition to vitamin D, vitamin E has demonstrated potential benefits in enhancing female fertility, particularly due to its antioxidant properties [87]. A deficiency in vitamin E has often been linked to recurrent pregnancy loss, highlighting its importance in maintaining a healthy pregnancy [88]. Some studies investigating the effects of vitamin E supplementation in women with unexplained infertility have produced mixed results. While some studies failed to show significant improvements in pregnancy rates, others suggest that vitamin E may help improve the endometrial environment and increase endometrial thickness, particularly in women experiencing implantation failure [84].

Vitamin E levels in both follicular fluid and serum have been associated with oocyte maturation, particularly in women undergoing IVF [89]. Studies suggest that optimal vitamin E levels in follicular fluid (ranging from 0.35 to 2 mg/dL) are crucial for proper oocyte maturation, and higher concentrations (10–15 mg/dL) are linked to improved embryo quality. Based on these findings, vitamin E supplementation (400 IU twice daily) may enhance outcomes in ART by improving oocyte quality and supporting embryo development [90].

Beyond vitamins D and E, other dietary components have shown promising effects in improving fertility, particularly in women with PCOS. One such component is green cardamom, a spice from the Zingiberaceae family. Green cardamom is rich in polyphenols and flavonoids, which possess potent antioxidant, antiinflammatory, and antibacterial properties [91,92]. Research suggests that administering 3 g/day of green cardamom to obese women with PCOS can improve key metabolic and endocrine markers, including hormonal balance and inflammatory cytokine levels. These benefits may, in turn, lead to improved reproductive outcomes for women with PCOS [93]. It is important to note that these findings have certain limitations. The study relies on self-reported data regarding diet and physical activity, involved slow patient recruitment due to strict eligibility criteria, was conducted in a single center, and faced challenges with patient non-compliance toward the end of the trial. This research investigates the effects of green cardamom on blood glucose levels, lipid profile, inflammatory markers, and blood pressure in obese individuals with non-alcoholic fatty liver disease, suggesting that improvements in these factors could positively influence fertility. However, these results should be interpreted with caution, and further studies are necessary to confirm or challenge these findings.

Alpha-lipoic acid (ALA), a naturally occurring antioxidant found in foods such as potatoes, broccoli, spinach, and red meat, also shows promise in the treatment of PCOS. ALA has anti-inflammatory, antioxidant, immunomodulatory, and insulin-sensitizing effects, which could be particularly beneficial for women with PCOS who experience insulin resistance. ALA is also an enzymatic cofactor in the mitochondrial respiratory chain, with the ability to enhance insulin sensitivity. It can eliminate reactive oxygen and nitrogen species both *in vivo* and *in vitro*, regenerating essential antioxidant molecules, such as coenzyme Q10, vitamin C, and vitamin E, and repairing proteins, lipids, and DNA damaged by oxidative stress. Some studies suggest that ALA supplementation, particularly when combined with inositol, may reduce symptoms of PCOS and improve biochemical markers. However, the effects on reproductive hormones remain unclear, and further research is needed to establish its full potential [40]. Suggested dosages for ALA vary, with some studies recommending 600 mg twice daily and others suggesting 800 mg per day [94].

It has long been evident that inositol is involved in the transduction of various endocrine signals, including insulin, thyroid hormones, gonadotropins, and prostaglandins. The modulatory role of inositol in glucose and insulin metabolism alone does not fully account for its clinical efficacy. Emerging evidence indicates that d-chiro-inositol directly regulates the expression of steroidogenic enzyme genes in human granulosa cells; moreover, d-chiro-inositol has been shown to increase testosterone levels in theca cells of women with PCOS [82]. Myo-inositol is believed to enhance ovarian function and fertility. However, the impact of myo-inositol on pregnancy rates remains uncertain, especially in women undergoing IVF. Randomized trials comparing myo-inositol with other antioxidants, insulin-sensitizing agents, and ovulation-inducing medications are necessary to determine its role in infertility treatment [95].

Looking forward, the use of nutraceutical supplements in treating infertility, particularly in overweight women with PCOS, holds significant promise. These supplements are accessible, affordable, and generally considered safe, making them an attractive option for many patients. However, future research must focus on identifying the most effective nutraceuticals and determining their optimal dosages to improve fertility outcomes. More randomized controlled trials are needed to establish definitive guidelines for their use in clinical practice, ensuring that these supplements are used effectively and safely in infertility treatments.

While nutraceutical supplementation holds promise for improving fertility outcomes in overweight infertile patients, particularly those with PCOS [96,97], further studies are essential to fully understand the underlying mechanisms and to establish clear treatment protocols. The combination of vitamins, antioxidants, and other dietary components could potentially provide a comprehensive approach to enhancing fertility, but continued research is necessary to refine these strategies.

The analysis of the scientific studies presented here outlines a promising framework for the use of nutraceutical supplementation as an adjunctive strategy in managing infertility, especially in patients with PCOS and overweight. However, it is crucial to interpret these results with caution.

There are potential benefits: Vitamin D emerges as a key modulator of reproductive and metabolic mechanisms, with positive effects on insulin sensitivity, endometrial receptivity, and ovarian function. Antioxidants such as vitamin E and omega-3 fatty acids can counteract oxidative stress, a significant factor in infertility, and improve reproductive outcomes. Probiotics and synbiotics show potential in modulating the composition of the intestinal microbiota, with beneficial effects on body weight and testosterone levels in overweight patients with PCOS. Other nutraceuticals, such as green cardamom, ALA, and myoinositol, may offer additional advantages, although further studies are needed to confirm their efficacy and establish optimal dosages.

However, there are some limitations. Despite the promising results, defining optimal vitamin D levels and the dosages of other nutraceuticals for fertility requires further research. Additionally, the individual variability in response to nutraceutical supplementation highlights the need for a personalized approach. The potential interactions between nutraceuticals and pharmacological treatments also necessitate careful clinical evaluation. Future research should focus on the synergistic effects of these nutraceuticals, determining how they might complement or enhance the efficacy of other fertility treatments.

Interactions between nutraceuticals can be synergistic, such as the combination of soy isoflavones and vitamin D [98], which has demonstrated benefits for postmenopausal bone health, or antagonistic, as in the case of iron and calcium, which compete for intestinal absorption [99].

Other relevant interactions include St. John’s Wort (Hypericum perforatum) and oral contraceptives, which can result in serious clinical consequences, such as an increased risk of unintended pregnancy, since St. John’s Wort induces the CYP3A4 enzyme, thereby reducing the efficacy of hormonal contraceptives [100]. Likewise, melatonin may act synergistically with anxiolytic medications (e.g., benzodiazepines) [101], leading to enhanced sedative effects, which are clinically significant in the treatment of insomnia during pregnancy or in the postpartum period [102].

Additionally, nutraceuticals containing grapefruit or grapefruit-derived compounds may interfere with the metabolism of a wide range of pharmaceutical drugs, due to the inhibition of cytochrome P450 3A4 (CYP3A4) in the intestinal wall [103]. This can result in elevated plasma concentrations of co-administered drugs, enhancing their pharmacological or toxic effects. In gynecology and obstetrics, this interaction is particularly relevant for medications such as oral contraceptives [104], antihypertensives [105], or immunomodulators, commonly used in various treatment protocols. Therefore, grapefruit-containing supplements should be used with caution or completely avoided when potential drug-nutraceutical interactions are a concern.

In gynecological and obstetric practice, these interaction dynamics take on particular importance due to the delicate physiological phases involved – pregnancy, postpartum, hormonal imbalance, and fertility treatments [106]. For example, phytoestrogens can interfere with hormonal therapies or contraceptive methods [107]; probiotics taken concurrently with antibiotics may alter treatment efficacy for bacterial vaginosis; and sedative agents such as valerian must be used cautiously during pregnancy, given the lack of robust safety data.

From a medico-legal standpoint, it is essential that the use of nutraceuticals is carefully evaluated by healthcare providers, always framed as adjunctive rather than substitutive therapy, and appropriately documented in the medical record. This is especially important in complex scenarios such as ART or the management of chronic conditions in women of reproductive and post-reproductive age [108,109]. Inadequate prescription or failure to assess potential interactions may expose clinicians to legal liability, particularly in the absence of strong scientific evidence or in the case of predictable adverse outcomes [110].

Nutraceutical supplementation can represent a safe, cost-effective adjunctive option for improving fertility outcomes in patients with PCOS and overweight. The integration of nutraceuticals into clinical practice should be based on an evidence-driven, personalized approach to ensure the most effective results.

Moreover, open communication between doctors and patients is essential to evaluate individual needs, monitor potential side effects, and make necessary adjustments in treatment plans.

In summary, while nutraceutical supplementation shows great promise in improving fertility in patients with PCOS and overweight, further studies are needed to optimize its clinical use and refine clinical practices and treatment protocols. This includes a deeper understanding of the optimal dosages, potential interactions, and long-term effects. Additionally, a more comprehensive understanding of the metabolic aspects of PCOS, such as insulin resistance and hormonal imbalances, is crucial for tailoring nutraceutical interventions more effectively. Future advancements in the field may also benefit from the integration of emerging scientific methodologies and artificial intelligence [111], which could enable more personalized and evidence-based approaches to nutraceutical use in reproductive medicine.

## 5 Conclusions

In conclusion, infertility is a complex issue affecting many couples globally, with obesity, particularly in women with PCOS, being a significant contributing factor. Nutritional interventions, including nutraceutical supplementation, offer potential benefits in improving fertility in overweight and obese patients by addressing underlying metabolic and hormonal imbalances.

The clinical management of obesity-related infertility goes beyond simple caloric restriction. It involves a personalized nutritional plan tailored to each patient’s hormonal and metabolic profile. Nutraceuticals are employed in a targeted manner, often alongside pharmacological treatments or ART, to enhance fertility outcomes.

Among the most commonly used nutraceuticals is vitamin D, widely prescribed for patients with deficiencies, especially women with PCOS, as it improves insulin sensitivity, endometrial receptivity, and embryo quality, all crucial for implantation and pregnancy success. However, serum vitamin D levels in women undergoing IVF do not appear to influence clinical outcomes, and the effects of vitamin D3 supplementation on female fertility are still under investigation. Vitamin E, known for its antioxidant properties, is used to improve semen quality in men and support ovarian function in women. Omega-3 fatty acids are commonly recommended due to their anti-inflammatory effects and their role in enhancing sperm motility and oocyte quality, thus contributing to a more favorable reproductive environment. Myoinositol is arguably one of the most prescribed supplements for women with PCOS, due to its proven ability to restore ovulatory function and improve insulin sensitivity, thereby reducing hyperinsulinemia and promoting hormonal balance. ALA, another powerful antioxidant with insulin-sensitizing properties, is also frequently used in PCOS patients to reduce oxidative stress and improve metabolic parameters.

As reported in this narrative review, the most promising approaches involve nutraceuticals aimed at optimizing weight reduction or mitigating oxidative stress, with direct effects on the regulation of the menstrual cycle, particularly in overweight patients. Moreover, in the context of PCOS, it is essential to identify the specific phenotype and to select the most appropriate support through a tailored approach.

Drawing on our clinical experience, nutraceuticals may serve as adjuncts to pharmacological therapies, with their use grounded in the need to address nutritional deficiencies and to ensure the optimal intake of specific compounds. Such an approach may positively influence key fertility parameters, including hormonal balance, ovulation, oocyte quality, embryo development, and the likelihood of achieving a full-term pregnancy.

Moreover, we have observed in our clinical practice what may be described as a placebo effect in patients taking nutraceuticals. Their mood often appears positively influenced by the belief that a simple product, such as a nutraceutical, can have a significant beneficial effect on their condition. It remains unclear whether this response is primarily driven by the patients’ own convictions or possibly influenced by our approach as healthcare providers prescribing these supplements. We aim to further explore this phenomenon through future research, potentially by correlating clinical data with patient surveys.

While promising evidence supports the benefits of specific nutrients and supplements, further research is needed to establish clear, evidence-based guidelines for their use. Nevertheless, a diet rich in essential nutrients, combined with the appropriate use of nutraceuticals, could represent a valuable tool in managing infertility, providing a more holistic approach to improving reproductive health.

## 6 Limitations

This article aims to provide an overview of the current situation regarding the topic at hand, without delving too deeply into the specifics of each individual issue. The intention is to avoid an overly complex analysis that could make the reading and understanding too burdensome for the reader. However, while the article does not explore the various issues in depth, it strives to remain comprehensive and accessible, making it easily digestible for the scientific community. A more detailed examination of each issue raised could be the subject of future research. Furthermore, when interpreting the results, it is necessary to take some limitations into account. The sample sizes reported in the different studies are often variable, which may limit statistical power and the generalization of the conclusions. In addition, the included articles show considerable heterogeneity in terms of the types of nutraceuticals used, dosages, duration of treatment, and characteristics of the studied populations; such variability makes direct comparison of the results challenging. Another critical issue concerns potential sources of bias: in several studies, the methods of randomization and blinding were not clearly described, leading to a possible risk of selection or performance bias; moreover, the presence of publication bias cannot be excluded, with a higher likelihood of publication for studies showing positive rather than negative results. Taken together, these elements suggest that the data should be interpreted with caution, while acknowledging that the available evidence supports a growing interest in and a potential role for nutraceuticals in the management of obesity.
